# Progress Toward Rubella Elimination — Western Pacific Region, 2000–2019

**DOI:** 10.15585/mmwr.mm6924a4

**Published:** 2020-06-19

**Authors:** Jennifer K. Knapp, Kayla M. Mariano, Roberta Pastore, Varja Grabovac, Yoshihiro Takashima, James P. Alexander, Susan E. Reef, José E. Hagan

**Affiliations:** ^1^Global Immunization Division, Center for Global Health, CDC; ^2^Expanded Programme on Immunization, World Health Organization Western Pacific Regional Office, Manila, Philippines.

Rubella is the leading vaccine-preventable cause of birth defects. Rubella typically manifests as a mild febrile rash illness; however, infection during pregnancy, particularly during the first trimester, can result in miscarriage, fetal death, or a constellation of malformations known as congenital rubella syndrome (CRS), commonly including one or more visual, auditory, or cardiac defects (*1*). In 2012, the Regional Committee of the World Health Organization (WHO) Western Pacific Region (WPR)* committed to accelerate rubella control, and in 2017, resolved that all countries or areas (countries) in WPR should aim for rubella elimination^†^ as soon as possible (*2*,*3*). WPR countries are capitalizing on measles elimination activities, using a combined measles and rubella vaccine, case-based surveillance for febrile rash illness, and integrated diagnostic testing for measles and rubella. This report summarizes progress toward rubella elimination and CRS prevention in WPR during 2000–2019. Coverage with a first dose of rubella-containing vaccine (RCV1) increased from 11% in 2000 to 96% in 2019. During 1970–2019, approximately 84 million persons were vaccinated through 62 supplementary immunization activities (SIAs) conducted in 27 countries. Reported rubella incidence increased from 35.5 to 71.3 cases per million population among reporting countries during 2000–2008, decreased to 2.1 in 2017, and then increased to 18.4 in 2019 as a result of outbreaks in China and Japan. Strong sustainable immunization programs, closing of existing immunity gaps, and maintenance of high-quality surveillance to respond rapidly to and contain outbreaks are needed in every WPR country to achieve rubella elimination in the region.

## Immunization Activities

During 1970–2005, rubella vaccination in 11 WPR countries^§^ focused on preventing CRS by vaccinating adolescent females; this strategy did not prevent all CRS cases, and countries adopted universal infant immunization (Table 1). By 2000, 16 (44%) of the 36 WPR countries that report immunization data to WHO and the United Nations Children’s Fund (UNICEF) included RCV1 in the routine immunization schedule; by 2015, all 36 had introduced it. By 2019, 34 (94%) countries had included a second dose of rubella-containing vaccine (RCV2). WHO and UNICEF estimate national RCV1 coverage for 27 countries in the region, using annual government-reported survey and administrative data; for the remaining nine countries,^¶^ coverage data reported by the immunization program are used.

**TABLE 1:** Year of introduction, age at vaccination, and estimated coverage with the first and second doses of rubella-containing vaccine (RCV),* and number of confirmed rubella cases^†^ and incidence,^§^ by country/area— World Health Organization (WHO) Western Pacific Region, 2000, 2010, and 2019

Country/Area	Year of introduction		2019 RCV schedule, age		2000			2010			2019^¶^		
			1st dose	2nd dose	% Coverage		No. of cases (incidence)^§^	% Coverage		No. of cases (incidence)^§^	% Coverage		No. of cases (incidence)^§^
	RCV1	RCV2			RCV1	RCV2		RCV1	RCV2		RCV1	RCV2	
Australia**	1989	1992	12m	18m	91	NR^††^	323 (15)	94	88	42 (2)	NR^††^	94	22 (1)
Brunei**	1988	1996	12m	18m	99	95	1 (3)	94	93	1 (2)	97	98	1 (2)
Cambodia	2012	2013	9m	18m	NA^§§^	NA^§§^	NR^††^	NA^§§^	NA^§§^	85 (5)	104	93	30 (2)
China	2007	2010	8m	18m	NA^§§^	NA^§§^	NR^††^	62	62^¶¶^	43,117 (30)	99	99	32,568 (23)
Hong Kong (CH)**	1990	1996	12m	6y	100	99	2,388 (343)	95	99	38 (5)	NR^††^	97	48 (6)
Japan**	1989	2006	12m	5y	94	NA^§§^	3,123 (24)	94	97	89 (1)	97**^¶^**	93**^¶^**	2,306 (18)
Laos	2011	2017	9m	12m	NA^§§^	NA^§§^	NR^††^	NA^§§^	NA^§§^	31 (4)	89	63	14 (2)
Macau (CH)**	1990	1994	12m	18m	90	89	20 (37)	92	87	2 (3)	98	96	79 (122)
Malaysia**^,^***	2002	2002	9m	12m	NA^§§^	NA^§§^	NR^††^	95	95	104 (3)	97	87	111 (3)
Mongolia	2009	2009	9m	2y	NA^§§^	NA^§§^	1,550 (570)	97	95	11 (3)	98	98	5 (2)
New Zealand**^,†††^	1990	1992	15m	4y	85	NR^††^	26 (6)	91	86	2 (0)	92**^¶^**	90**^¶^**	1 (0)
Papua New Guinea	2015	2015	9m	18m	NA^§§^	NA^§§^	NR^††^	NA^§§^	NA^§§^	5 (1)	33	20	5 (1)
Philippines	2010	2015	9m	12m	NA^§§^	NA^§§^	NR^††^	10^§§§^	NA^§§^	1,440 (14)	73	68	198 (2)
Singapore**	1982	1990	12m	18m	96	98	312 (61)	95	96	158 (27)	95**^¶^**	84**^¶^**	7 (1)
South Korea	1983	1997	12–15m	4–6y	95	39	107 (2)	98	98	21 (0)	97	97	8 (0)
Vietnam	2015	NA^§§^	18m	NA^§§^	NA^§§^	NA^§§^	NR^††^	NA^§§^	NA^§§^	2,300 (24)	90**^¶^**	NA^§§^	69 (1)
**Pacific Island Countries and Territories**
American Samoa (US)	1980s	2003**^¶¶¶^**	12m	4y	90	94	0 (0)	77	65	NR^††^	NR^††^	NR^††^	NR^††^
Cook Islands (NZ)	2006	2006	15m	4y	NA^§§^	NA^§§^	0 (0)	99	98	0 (0)	99**^¶^**	99**^¶^**	0 (0)
Fiji**	2003	2004	12m	18m	NA^§§^	NA^§§^	NR^††^	94	94	0 (0)	94**^¶^**	94**^¶^**	NR^††^
French Polynesia (FR)**	2010	2010	12m	18m	NA^§§^	NA^§§^	NR^††^	99	84	0 (0)	98**^¶^**	98**^¶^**	NR^††^
Guam (US)	1980s	1998	12m	4–6y	93	94	0 (0)	NR^††^	NR^††^	0 (0)	82**^¶^**	83**^¶^**	0 (0)
Kiribati	2004	2007	12m	4y	NA^§§^	NA^§§^	0 (0)	89	21	0 (0)	84**^¶^**	79**^¶^**	0 (0)
Marshall Islands	1982	1998	12m	13m	93	6	0 (0)	97	90	0 (0)	85	64	0 (0)
Micronesia	1982	1995	12m	≥13m	85	50	NR^††^	80	75	NR^††^	78	52	0 (0)
Nauru	2006	2006	12m	15m	NA^§§^	NA^§§^	0 (0)	99	92	NR^††^	96	96	0 (0)
New Caledonia (FR)	1994	1994	12m	16m	NR^††^	NR^††^	NR^††^	99	78	NR^††^	96^¶^	92^¶^	NR^††^
Niue (NZ)**	1979	1998	15m	4y	99	99	0 (0)	99	99	0 (0)	100	100	0 (0)
Northern Mariana Islands (US)	1980s	1992	12m	4–6y	NA^§§^	NA^§§^	0 (0)	93	39	0 (0)	75	90	0 (0)
Palau	1986	1995	12m	15m	83	75	0 (0)	39	39	0 (0)	97	88	0 (0)
Samoa	2003	2005	12m	15m	NA^§§^	NA^§§^	NR^††^	56	30	0 (0)	96	59	0 (0)
Solomon Islands	2013	2018	12m	18m	NA^§§^	NA^§§^	NR^††^	NA^§§^	NA^§§^	0 (0)	81	55	0 (0)
Tokelau (NZ)	2003	2005	12m	15m	NA^§§^	NA^§§^	0 (0)	95	95	0 (0)	98	98	0 (0)
Tonga	2002	2002	12m	18m	NA^§§^	NA^§§^	0 (0)	86	84	0 (0)	99	100	NR^††^
Tuvalu	2005	2005	12m	18m	NA^§§^	NA^§§^	0 (0)	85	87	0 (0)	88**^¶^**	81**^¶^**	NR^††^
Vanuatu	2015	NA^§§^	12m	NA^§§^	NA^§§^	NA^§§^	NR^††^	NA^§§^	NA^§§^	NR^††^	76	NA^§§^	0 (0)
Wallis and Fortuna (FR)	NR^††^	NR^††^	12m	16m	NA^§§^	NA^§§^	4 (272)	NR^††^	NR^††^	NR^††^	105	125	NR^††^
Total Western Pacific Region****	—	—	—	—	**11**	**11**	**7,854 (36)**	**59**	**59**	**47,446 (25)**	**96**	**91**	**35,472 (18)**

**Abbreviations:** CH = China; FR = France; NA = not applicable; NR = not reported; NZ = New Zealand; RCV1 = first RCV dose; RCV2 = second RCV dose; RI = routine immunization; UNICEF = United Nations Children's Fund; US = United States.

* Based on data from WHO-UNICEF Estimates of National Immunization Coverage, WHO/UNICEF Joint Reporting Form, or WHO Western Pacific Regional Office databases. https://www.who.int/immunization/monitoring_surveillance/data/en

^†^ Includes cases confirmed by laboratory testing or epidemiologic linkage, as reported in the WHO/UNICEF Joint Reporting Form or other WHO Western Pacific Regional Office databases or reports. https://www.who.int/immunization/monitoring_surveillance/data/en

^§^ Per million population.

^¶^ 2019 data are as of May 14, 2020; for countries without RCV1 and RCV2 estimates by this date, 2018 coverage values are used.

** Initial rubella vaccination strategy involved vaccination of adolescent females to prevent congenital rubella syndrome in the following countries/areas, years, and age groups: Australia (1971–1994, 12–14 years); Brunei (1978–1995, 12–13 years); Fiji (1975–2005, 11–14 years); French Polynesia (France) (1990s, 10 years); Hong Kong (China) (1978–1995, 11 years); Japan (1977–1995, 12–15 years); Macau (China) (1987–2002, 10–13 years); Malaysia (1987–2008, 12 years); New Zealand (1979–1991, 11 years); Niue (New Zealand) (late 1970s, 11–12 years); Singapore (1976–1982, 11–12 years); and South Korea (1994–2001, 16 years).

^††^ Not reported because country did not report coverage or cases in the year specified.

^§§^ Not applicable because dose was not included in the vaccination schedule for that year.

^¶¶^ RCV2 coverage as described by Su Q, Ma C, Wen N, et al. https://www.sciencedirect.com/science/article/pii/S0264410X18303499?via%3Dihub.

*** 2018 RCV schedule includes an additional dose given at age 7 years.

^†††^ Rubella vaccination of children aged 4 years during 1970–1978, then switch to adolescent female vaccination during 1979–1991.

^§§§^ RCV2 coverage as described by Lopez AL, Raguindin PFN, Silvestre MA, Fabay XCJ, Vinarao AB, Manalastas R. https://www.hindawi.com/journals/ijpedi/2016/8158712/.

^¶¶¶^ Approximate year of introduction.

***Regional average coverage and incidence are calculated for the countries reporting information. For coverage if a rubella vaccine was not in the vaccination schedule (NA) a value of zero was used, and the country included in the denominator.

Population immunity of ≥85% is needed to achieve herd immunity and interrupt endemic rubella virus transmission (*1*). Regional RCV1 coverage increased from 11% in 2000 to 96% in 2019 and has been ≥90% since 2015 because of vaccine introduction and achievement of high vaccination coverage in China (2007) and Vietnam (2015) (Figure). In 2019, 24 (67%) countries achieved ≥90% RCV1 coverage, and 19 (53%) countries achieved ≥90% coverage for RCV1 and RCV2 (Table 1). However, two countries and six islands did not reach 85% RCV1 coverage, leaving 793,850 infants unprotected.

**FIGURE:**
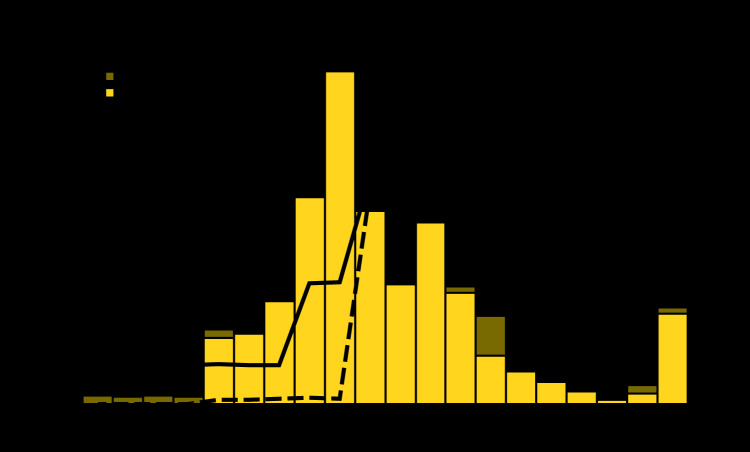
Confirmed rubella cases,* by year of rash onset and country,^†^ and estimated regional coverage with first and second doses of rubella-containing vaccine^§^ — World Health Organization (WHO) Western Pacific Region, 2000–2019 **Abbreviations:** RCV1= first dose of a rubella-containing vaccine; RCV2 = second dose of a rubella-containing vaccine. *Confirmed rubella cases reported by countries and areas to WHO. A case of rubella was laboratory-confirmed when rubella-specific immunoglobulin M antibody was detected in serum, rubella-specific RNA was detected by polymerase chain reaction testing, or rubella virus was isolated in cell culture in a person who had not been vaccinated in the 30 days before rash onset; a case of rubella was confirmed by epidemiologic linkage when a case of febrile rash illness was linked in time and place to a laboratory-confirmed rubella case. ^†^ The following countries began reporting rubella surveillance data after 2000: China (2004), Vietnam (2005), Cambodia (2006), Laos (2007), Papua New Guinea (2007), and Malaysia (2010). ^§^ WHO and United Nations Children’s Fund Estimates of National Immunization Coverage, July 15, 2019. https://www.who.int/immunization/monitoring_surveillance/data/en/.

During 1970–2019, 84.3 million persons were vaccinated during 62 SIAs conducted in 27 countries (weighted regional coverage = 81%) (Table 2) (*4*–*8*). Reported administrative coverage was ≥95% in 30 (50%) of 60 SIAs with available data.

**TABLE 2:** Characteristics of nationwide rubella supplementary immunization activities (SIAs),* by year and country/area — World Health Organization (WHO) Western Pacific Region, 1970–2019^†^

Country/Area	Year	RCV used	SIA type	Age group targeted	Population reached in targeted age group no. (%)
American Samoa (US)	2019	MMR	M–outbreak	6m–adults	12,932 (41)
Australia	1998	MMR	Catch-up	1–3.5y	60,028 (37)
				5–12y	1,333,980 (75)
Brunei	2008–2009	MMR	Catch-up	3–6y	27,161 (98)
Cambodia	2013	MR	Catch-up	9m–14y	4,576,633 (105)^§^
	2016	MR	M–outbreak	9m–4y	766,743 (91)
	2017	MR	Follow-up	6m–4y	1,451,821 (90)^¶^
Cook Islands (NZ)	2006	MR	Catch-up	1–15y F: 16–35y	5,829 (90)
Fiji	2006	MR	M–outbreak	6m–4y	89,747 (98)
	2017	MR	Catch-up	1–11y	178,069 (95)
	2019	MR	M–outbreak	6m–4y 19y–39y	85,911 (100) 257,566 (94)
Hong Kong (CH)	1997	MMR	Catch-up	19–39y	1,100,464 (77)
Kiribati	2006	MR	Catch-up	1–14y F: 15–19y	40,568 (95)
	2009	MMR	Follow-up	1–4y	9,865 (107)^§^
	2013	MR	Follow-up	1–4y	1,700 (85)
	2019	MR	Catch-up	1–14y	42,838 (107)^§^
Laos	2011	MR	M–outbreak	9m–19y	2,614,002 (97)
	2014	MR	M–outbreak	9m–9y	1,569,224 (100)
	2017	MR	Follow-up	9m–4y	703,924 (100)
	2019	MR	M–outbreak	6m–9y	937,064 (60)
Malaysia	1987–1989	Rubella	Catch-up	F: 15–44y	NR (62)
Marshall Islands	2002	MMR	Follow-up	1–4y	4,383 (77)
	2003	MMR	M–outbreak	6m–40y	37,111 (91)
	2019	MR	M–outbreak	1–5y	NR (79)
Micronesia	2014	MMR	M–outbreak	6m–49y	71,388 (87)
Mongolia	2012	MR	Catch-up	3–14y	522,429 (93)
	2016	MR	M–outbreak	18–30y	549,846 (88)
	2019	MR	Catch-up	10–18y	400,961 (96)
New Zealand	1970	Rubella	Catch-up	5–9y	NR (95)
	1997	MMR	M–outbreak	2–10y	474,022 (75)
	2001	MMR	Catch-up	5–10y	NR (NR)
Niue (NZ)	2003	MMR	Catch-up	5–11y	100 (36)
Northern Mariana Islands (US)	2002	MMR	Follow-up	1–6y	438 (35)
	2018	MR	Catch-up	1–18y	36,175 (74)
	2019	MR	Catch-up	19–62y	NR (74)
Papua New Guinea	2015–2016	MR	M–outbreak	6m–15y	1,238,290 (63)
	2019	MR	Follow-up	6m–4y	1,180,422 (101)^§^
Philippines	2011	MR	M–outbreak	9m–8y	15,649,907 (84)
	2014	MR	M–outbreak	9m–4y	10,402,489 (91)
	2018	MMR	M–outbreak	6m–4y	4,982,898 (46)
	2019	MMR	M–outbreak	5–12y	2,457,514 (29)
Samoa	2003	MR	R–outbreak	1–18y	47,448 (88)
				F: 19–49y	19,730 (103)^§^
	2005	MR	Follow-up	9m–2y	11,610 (86)
	2008	MR	Follow-up	9m–4y	22,864 (91)
	2009	MR	Disaster	6m–4y	21,142 (76)
	2017	MR	M–outbreak	1–12y	57,229 (95)
	2019	MR	M–outbreak	6m–50y	187,369 (93)
Singapore	1997	MMR	M–outbreak	12–18y	NR (NR)
	2013	MMR	Catch-up	6–7y	38,436 (95)
Solomon Islands	2012	MR	R–outbreak	1–4y	67,106 (101)^§^
	2014	MR	M–outbreak	6m–29y	394,584 (105)^§^
	2019	MR	M–outbreak	6m–5y	87,855 (99)
South Korea	2001	MR	M–outbreak	8–16y	5,614,327 (96)
	2006–2009	MMR	Follow-up	8y	2,205,333 (99)
Tokelau (NZ)	2003	MMR	R–outbreak	1–15y F: CBA**	838 (98)
Tonga	2002	MR	R–outbreak	1–13y	37,279 (95)
				F: 14–40y	18,321 (95)
	2019	MR	M–outbreak	6m–24y	54,590 (94)
Tuvalu	2005	MR	Catch-up	1–34y	5,469 (96)
	2010	MR	Follow-up	1–5y	1,095 (79)
Vanuatu	2013	MR	Follow-up	1–4y	33,604 (102)^§^
	2015	MR	Catch-up	1–15y	103,676 (103)^§^
Vietnam	2014–2015	MR	Catch-up	1–14y	19,735,753 (98)
	2016	MR	Catch-up	16–17y	1,787,588 (95)
**Total Western Pacific Region**	**1970–2019**	**—**	**—**	**—**	**84,339,251 (81)**

**Abbreviations:** CBA = childbearing age; CH = China; F = female; FR = France; m = months; M-outbreak = measles outbreak; MMR = measles, mumps, and rubella vaccine; MR = measles and rubella vaccine; NR = not reported; NZ = New Zealand; R-outbreak = rubella outbreak; RCV = rubella-containing vaccine; SIA = supplemental immunization activity; US = United States; y = years.

* Rubella SIAs use a combined measles-rubella vaccine; these SIAs generally use two target age ranges: 1) initial, nationwide catch-up SIAs target all children aged 9 months–14 years, with the goal of eliminating susceptibility to rubella virus in the general population, and 2) follow-up nationwide SIAs generally conducted every 2–4 years target children not included in the previous SIA, who are generally aged 9–59 months (their goal is to protect children who did not respond to the first measles vaccine dose and to provide another opportunity for vaccination). Rubella SIAs also occur as a result of measles outbreak response SIAs when MR or MMR is used for the campaign. The exact age range for follow-up or outbreak SIAs depends on the age-specific incidence of measles, coverage with vaccine containing measles and rubella through routine services, and the time since the last SIA.

^†^ SIAs conducted in 2019 might display interim rather than final numbers of persons vaccinated.

^§^ Values >100% indicate that the intervention reached more persons than the estimated target population. The numerator was the total children vaccinated, and the denominator was the estimated target calculated for vaccination.

^¶^ A post-campaign coverage survey estimated that 75% of children within the targeted ages were vaccinated.

** The SIA denominator indicates that >15 birth cohorts were targeted during this rubella outbreak response; it is expected that, similar to what was found for SIAs on other islands with rubella outbreaks at that time, the additional vaccine recipients were women of childbearing age.

## Surveillance Activities

Case-based measles and rubella surveillance data are requested monthly by WHO from all WPR countries. Most countries** use an acute fever and maculopapular rash case definition to begin a case investigation and laboratory testing. Some countries also report national or sentinel CRS surveillance data. Rubella cases are confirmed by serology or virus detection or an epidemiologic link to a laboratory-confirmed case. Suspected CRS cases can also be clinically^††^ confirmed. The WHO Global Measles and Rubella Laboratory Network has supported laboratory confirmation and genotyping since 2005. Indicators of combined measles and rubella surveillance performance include 1) the number of febrile rash illness cases discarded as neither measles nor rubella (target: ≥2 per 100,000 population); 2) the percentage of cases with adequate investigations that include all essential data elements^§§^ (target: ≥80%); 3) the percentage of cases with adequate blood specimens collected within 28 days of rash onset (target: ≥80%, excluding epidemiologically linked cases); and 4) the percentage of specimens with laboratory results reported within 4 days after receipt in the laboratory (target: ≥80%).

The number of WPR countries reporting rubella data increased from 22 (61%) in 2000 to 29 (81%) in 2019 (Table 1). Five countries,^¶¶^ representing 11% of the regional population, have implemented nationwide CRS surveillance; another seven*** (7% of the population) conduct sentinel surveillance; and four countries^†††^ (82% of the population) and the 21 countries included in the Pacific Islands Countries and Territories (<1% of the population) do not conduct CRS surveillance. During 2010–2018, the median regional nonmeasles/nonrubella discard rate was 3.0 per 100,000 population, ranging from 1.7 (2010) to 9.8 (2018). From 2010 to 2018, the percentage of suspected measles/rubella cases with adequate investigations increased from 76% to 84% and the percentage with adequate blood specimens collected increased from 71% to 82%; the percentage of specimens with laboratory results increased from 48% within 7 days to 76% within 4 days. Regional surveillance indicators are near the target values and all appear to have improved in response to measles outbreaks in 2018.

## Rubella Incidence, Outbreaks, and Genotypes

During 2000–2008, regional rubella incidence increased from 35.5 cases per million population to a peak of 71.3, following initiation of national surveillance in China and Vietnam. Following RCV1 introduction in China, Vietnam, and 18 other countries during 2000–2015, rubella incidence decreased to a historic low of 2.1 per million in 2017 but increased to 18.4 in 2019 (Figure). China, the most populous country, has reported 88% of regional rubella cases since it began reporting in 2004. Nationwide outbreaks occurred in Hong Kong (2000), the Philippines (2001, 2010, and 2017), Samoa (2003), Tokelau (2003), Mongolia (2007), Fiji (2011), Vietnam (2011), Japan (2012–2013),^§§§^ Tonga (2002) (Angela Merianos, WHO Pacific Health Security and Communicable Diseases, personal communication, December 2019), and the Solomon Islands (2012) (*8*). The regional rubella resurgence in 2018–2019 (Figure) was driven by transmission among susceptible males aged 30–55 years in Japan (2018–2019) and among unvaccinated adolescents and young adults in China (2019), with spread to other age groups that included pregnant women. These two outbreaks, which involved rubella virus importations from >15 other countries, accounted for 98% of regional rubella cases in 2018–2019. Only a few countries (Japan, Solomon Islands, and Vietnam) identified CRS cases that occurred after outbreaks. Since 2010, three rubella virus genotypes (1E, 2B, and 1J) have been detected in the region. Genotypes 1E and 2B have broad, annual circulation within the region. Genotype 1J was detected in four WPR countries before 2013, but not since.

## Regional Verification of Rubella Elimination

The Western Pacific Regional Committee (*1*) urged countries to submit measles elimination progress reports for review by the Regional Verification Commission in 2013; verification guidelines were revised in 2017 to include verification of rubella elimination (*1*). As of September 2019, five of seventeen (29%) countries^¶¶¶^ (Australia, Brunei, Macau, New Zealand, and South Korea) have been verified to have achieved and sustained rubella elimination (*9*).

## Discussion

Following the 2012 WHO Regional Committee resolution for rubella control, introduction of combined measles and rubella vaccine accelerated, and nearly all countries in WPR now include 2 RCV doses in the routine immunization program. Regional coverage is high, and rubella incidence declined to a historic low in 2017.

Despite high regional coverage, variation in immunity exists among and within countries. Eight countries were unable to reach protective herd immunity of 85% in their 2018 birth cohorts, perpetuating immunity gaps among children. Recent success achieving high coverage also masks susceptibility among older persons. In WPR, immunity gaps developed from historical adolescent female vaccination programs and by introduction of rubella vaccine in the childhood immunization program without vaccinating those who were not age-eligible according to the childhood vaccination schedule at the time of introduction. As long as immunity gaps persist, countries remain vulnerable to importations, outbreaks that include adults, and CRS-affected pregnancies. Lack of coordination toward elimination among countries and regions creates an inequitable strain on achieving and maintaining rubella elimination because of importations via travel and transit.

Strategies to close identified immunity gaps vary by country. Japan is targeting adult males, testing for immunity and vaccinating susceptible persons. Vietnam annually targets children in a portion of districts determined to be at high risk. Other countries have incidentally boosted immunity to rubella by conducting SIAs in response to measles outbreaks, using combined measles-rubella or measles-mumps-rubella vaccine, although rarely in response to rubella outbreaks.

The World Bank classifies 10 countries in the region as low-middle income,**** allowing some opportunities for external support for the routine immunization program, targeted immunization activities, and outbreak response support. However, external immunization funding is not currently well-aligned with strategies to achieve a regional elimination goal. The remaining countries must self-finance rubella elimination, given the absence of a broad mechanism for external immunization funding support in middle income countries. In addition, many countries use domestic vaccine suppliers that set vaccine prices and whose production capacity might not meet outbreak response needs. Five countries have been verified as having eliminated endemic rubella transmission; however, other countries with a long history of rubella vaccination and surveillance and with a low annual incidence might also have achieved elimination but have not yet requested verification.

The findings in this report are subject to at least three limitations. First, sensitivity of integrated measles and surveillance for rubella is low because it is a milder illness, resulting in underdetection of cases. Second, direct comparisons among countries might not be valid because of variations in capacity for case investigation and laboratory testing, the monitoring of progress toward elimination, level and source of financing, and the priority given to closing immunity gaps. Finally, the region has countries with widely disparate population sizes, and regional trends might obscure challenges or successes in less populous countries.

The participation of all WPR countries will be needed to attain regional rubella elimination and prevent the devastating consequences of rubella infection during pregnancy. Efforts to achieve these goals include sustaining high population immunity, identifying and addressing existing immunity gaps, and maintaining high-quality surveillance to allow for rapid outbreak detection and prompt response to contain outbreaks.

**Summary**

**What is already known about this topic?**

Before 2000, 16 countries and areas in the Western Pacific Region (WPR) included rubella-containing vaccine (RCV) in the infant immunization program; three more vaccinated adolescent females only.

**What is added by this report?**

All of WPR’s 37 countries and areas have introduced RCV in the infant immunization program, achieving 96% regional coverage. Rubella incidence declined to 2.1 cases per million population in 2017 but increased again because of outbreaks in groups with low immunity.

**What are the implications for public health practice?**

WPR has made rapid progress toward rubella elimination and prevention of congenital rubella syndrome since 2010. The 2018–2019 resurgence demonstrates that immunity gaps remain among adolescents and adults; if these are addressed, regional rubella elimination could be rapidly achieved.

## Footnotes

* The Western Pacific Region, one of the six regions of the World Health Organization, consists of 37 countries and areas with a population of approximately 1.9 billion, including American Samoa (United States), Australia, Brunei, Cambodia, China, Cook Islands (New Zealand), Micronesia, Fiji, French Polynesia (France), Guam (United States), Hong Kong (China), Japan, Kiribati, Laos, Macau (China), Malaysia, Marshall Islands, Mongolia, Nauru, New Caledonia (France), New Zealand, Niue (New Zealand), Northern Mariana Islands (United States), Palau, Papua New Guinea, Philippines, Pitcairn Islands (United Kingdom), Samoa, Singapore, Solomon Islands, South Korea, Tokelau (New Zealand), Tonga, Tuvalu, Vanuatu, Vietnam, and Wallis and Futuna (France).

† Rubella elimination is defined as the absence of endemic rubella virus transmission in a defined geographical area (e.g., region or country) for ≥12 months in the presence of a well-performing surveillance system.

§ Initial rubella vaccination strategy involved vaccination of adolescent females to prevent CRS in the following countries and areas, years, and age groups: Australia (1971–1994, 12–14 years); Brunei (1978–1995, 12–13 years); Fiji (1975–2005, 11–14 years); French Polynesia (France) (1990s, 10 years); Hong Kong (China) (1978–1995, 11 years); Japan (1977–1995, 12–15 years); Macau (China) (1987–2002, 10–13 years); Malaysia (1987–2008, 12 years); New Zealand (1979–1991, 11 years); Niue (New Zealand) (late 1970s, 11–12 years); Singapore (1976–1982, 11–12 years); and South Korea (1994–2001, 16 years).

¶ The WHO/UNICEF estimates of national immunization coverage are not calculated for nine areas in the following countries: China (Hong Kong and Macau), France (French Polynesia, New Caledonia, and Wallis and Futuna), New Zealand (Tokelau), and United States (American Samoa, Guam, and Northern Mariana Islands). The Pitcairn Islands (United Kingdom), with a population of <100 persons, does not report to WHO/UNICEF and is excluded from all calculations.

** As of 2019, 32 countries use a case definition of acute fever and maculopapular rash to identify suspected cases of both rubella and measles, leading to laboratory testing for both diseases. The other four countries have separate surveillance systems for rubella and measles. In those countries, a clinician’s diagnosis is based on rubella signs and symptoms (described as maculopapular rash and fever [if measured] and either arthritis/arthralgia or lymphadenopathy) and no testing for measles is done; clinically diagnosed rubella cases are not included in the regional surveillance performance indicators.

†† CRS can be clinically confirmed in an infant when a qualified physician detects at least two of the complications listed in group A (cataract or cataracts, congenital glaucoma, congenital heart disease, hearing impairment, or pigmentary retinopathy), or one in group A and one in group B (purpura, splenomegaly, microcephaly, developmental delay, meningoencephalitis, radiolucent bone disease, or jaundice within 24 hours after birth).

§§ Essential data elements include name or identifier, date of birth or age, sex, place of residence, vaccination status or date of last vaccination, date of rash onset, date of notification, date of investigation, date of specimen collection, and place of infection or travel history.

¶¶ Countries and areas with nationwide surveillance for congenital rubella syndrome include Australia, Hong Kong (China), Japan, New Zealand, and South Korea.

*** Countries and areas with sentinel-site surveillance for congenital rubella syndrome include Brunei, Cambodia, Laos, Macau (China), Papua New Guinea, Singapore, and Vietnam.

††† Countries with no CRS surveillance include China, Malaysia, Mongolia, and Philippines.

§§§ https://apps.who.int/immunization_monitoring/globalsummary/timeseries/tsincidencerubella.html.

¶¶¶ The Regional Verification Commission reviews measles and rubella elimination reports from 17 units: each of 14 WHO member states, two Chinese Special Administrative Regions (Hong Kong and Macau), and the subregion of the Pacific Island Countries and Territories. The Pacific Islands Countries and Territories are reviewed as a single epidemiologic unit; they include American Samoa (United States), Cook Islands, Fiji, French Polynesia (France), Guam (United States), Kiribati, Marshall Islands, Micronesia, Nauru, New Caledonia (France), Niue (New Zealand), Northern Mariana Islands (United States), Palau, Samoa, Solomon Islands, Tokelau (New Zealand), Tonga, Tuvalu, Vanuatu, and Wallis and Futuna (France).

**** Cambodia, Kiribati, Laos, Micronesia, Mongolia, Papua New Guinea, Philippines, Solomon Islands, Vanuatu, and Vietnam.
